# Supplementary material for: Geographic Location Affects the Bacterial Community Composition and Diversity More than Species Identity for Tropical Tree Species
Source: Plants (Basel). 2024 Jun 5;13(11):1565. doi: 10.3390/plants13111565 (PMC11175100; doi:10.3390/plants13111565)
Supplement: Supplementary file 1 [file plants-13-01565-s001.zip › plants-3017573-supplementary.pdf]

Geographic location affects the bacterial community composition more than species identity in tropical tree species

Kepeng Ji[a,c†] , Yaqing Wei[a,b†], Guoyu Lan[a,b*]

The following supporting information is available for this article:

Supplementary figures

Figure S1 Study sites on Hainan Island. Red and blue solid circles each represent *V. mangachapoi* and *D. pectinatum* on the map.

Figure S2 Sampling design on Hainan Island. For each plot, we selected three trees for sampled root (yellow solid circles) and leaves (green solid circles) from all four cardinal directions.

Figure S3 Environmental variables in leaf physicochemical properties of *D. pectinatum and V. mangachapoi* among different sampling sites. The significant differences between species and sites were detected by adonis.

Figure S4 Environmental variables in soil physicochemical properties of *D. pectinatum* and *V. mangachapoi* among different sampling sites. The significant differences between species and sites were detected by adonis.

Figure S5 Bacterial community composition at class level of *D. pectinatum* and *V. mangachapoi*.

Figure S6 The biomarker taxa of bacterial communities in *D. pectinatum* and *V. mangachapoi*. A: LEfSe identifying the biomarker taxa associated with *D. pectinatum* and *V. mangachapoi* (class level). B: Differences in bacterial composition in different geographical locations. *, $P < 0.05$; **, $P < 0.01$, ***, $P < 0.001$.

Figure S7 Redundancy analysis ordination of the leaf and soil samples, based on the bacterial community compositions (OTU level). *, $P < 0.05$; **, $P < 0.01$, ***, $P < 0.001$. A: Leaf sample. B: Root sample.

Supplementary tables

Table S1 Analysis of variance model results on the effects of geographical locations,

plant compartments and plant identity on α-diversity (OTU richness) of bacteria in *D. pectinatum* and *V. mangachapoi*.

Table S2 Permuted multivariate analysis of variance (PERMANOVA) tables for differences in bacterial community compositions (OTU level).

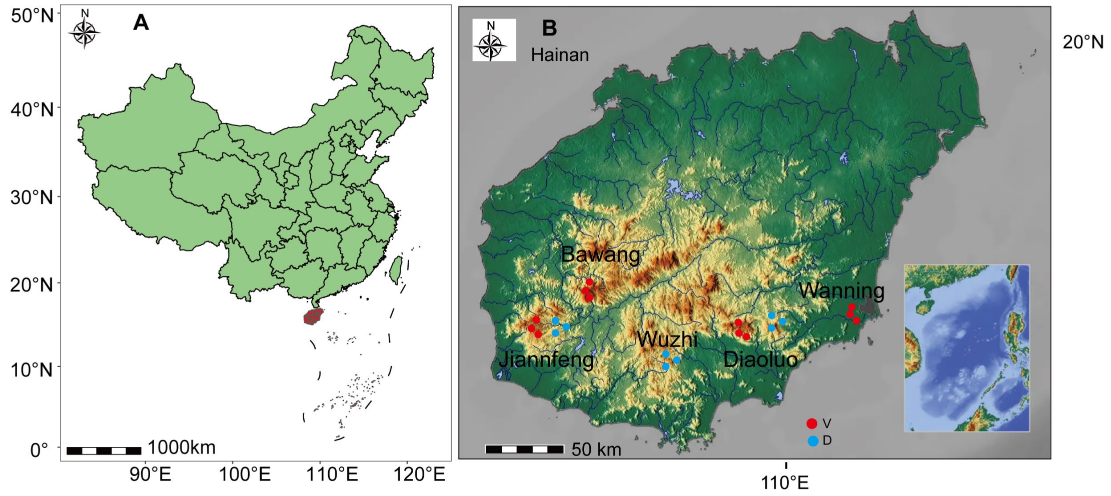

Figure S1 Study sites on Hainan Island. Red and blue solid circles each represent *V. mangachapoi* and *D. pectinatum* on the map.

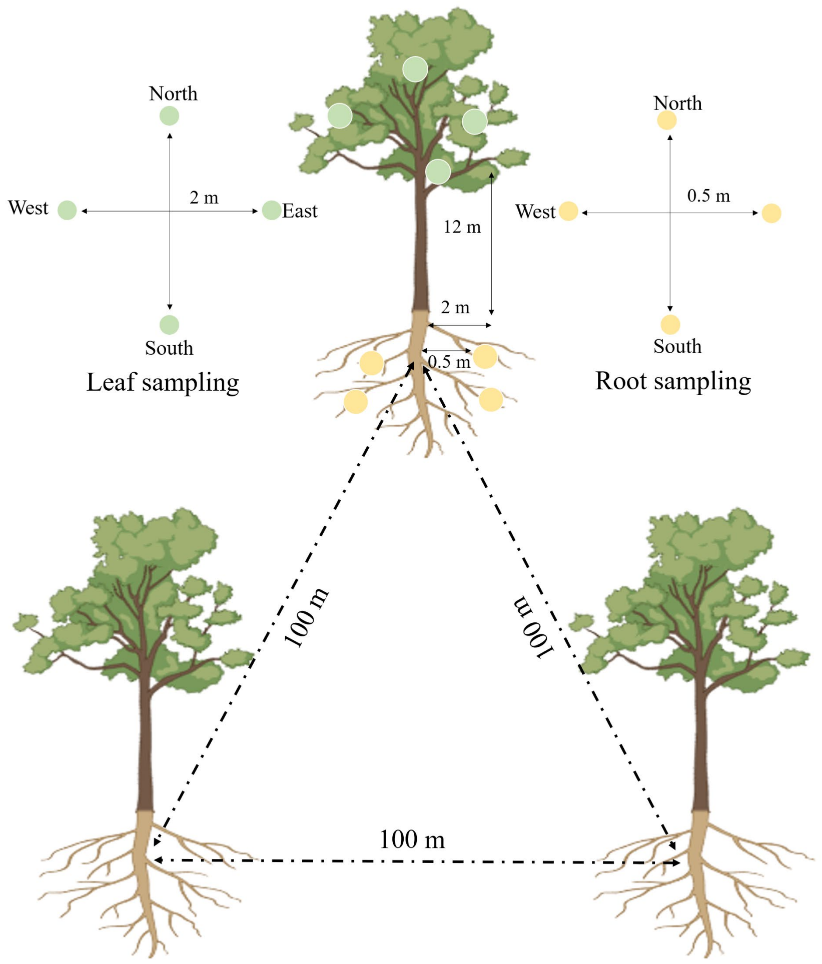

Figure S2 Sampling design on Hainan Island. For each plot, we selected three trees for sampled root (yellow solid circles) and leaves (green solid circles) from all four cardinal directions.

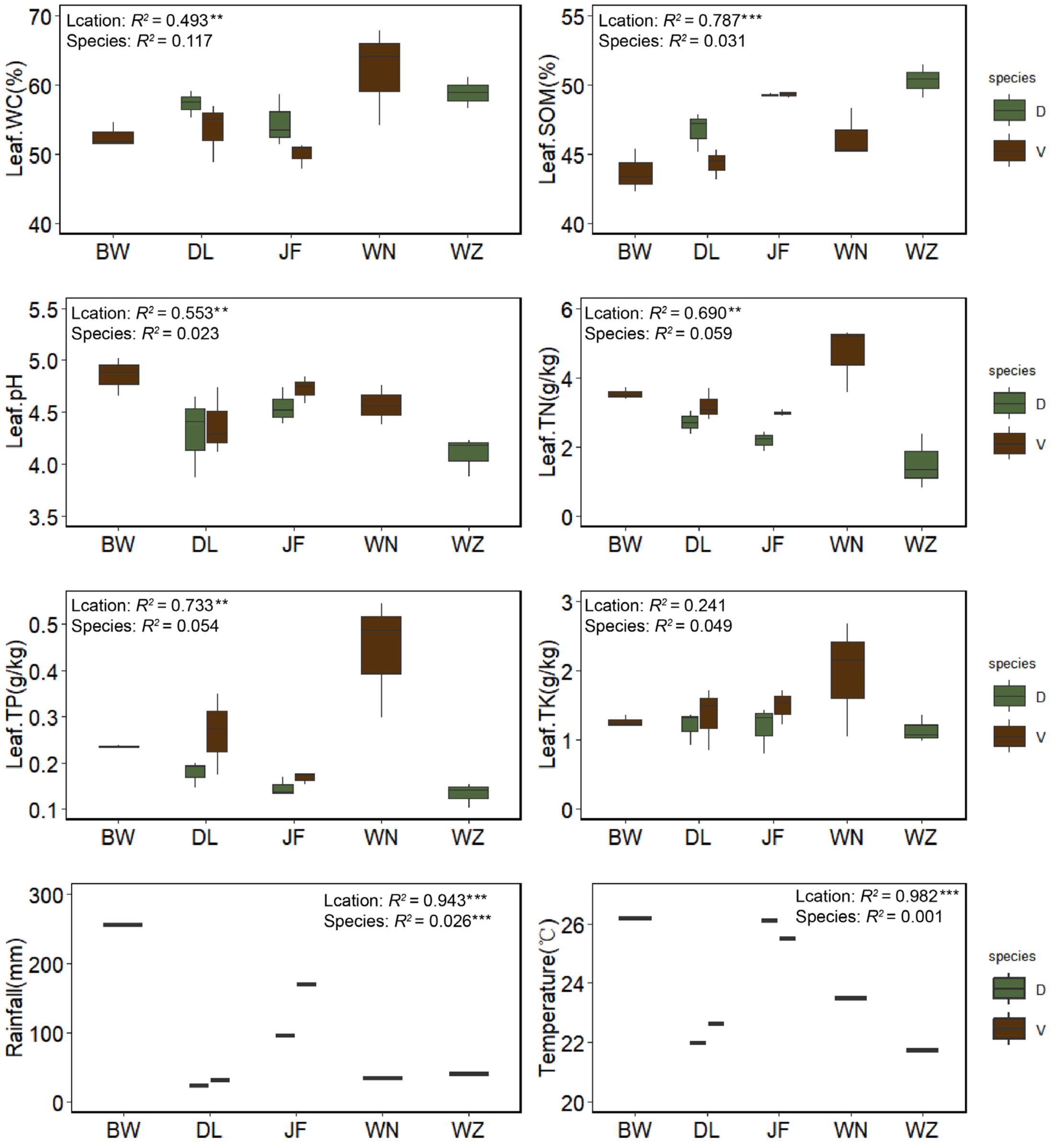

Figure S3 Environmental variables in leaf physicochemical properties of *D. pectinatum and V. mangachapoi* among different sampling sites. The significant differences between species and sites were detected by adonis. SOM: organic matter, TN: Total nitrogen, TP: Total phosphorus, TK: Total potassium, WC: Water content, Rainfall: Mean monthly rainfall, Temperature, mean monthly temperature. D, *Dacrydium pectinatum*; V, *Vatica mangachapoi*; DL, Diaoluo; JF, Jianfeng; WZ, Wuzhi; BW, Bawang; WN, Wanning. *, $P < 0.05$; **, $P < 0.01$, ***, $P < 0.001$.

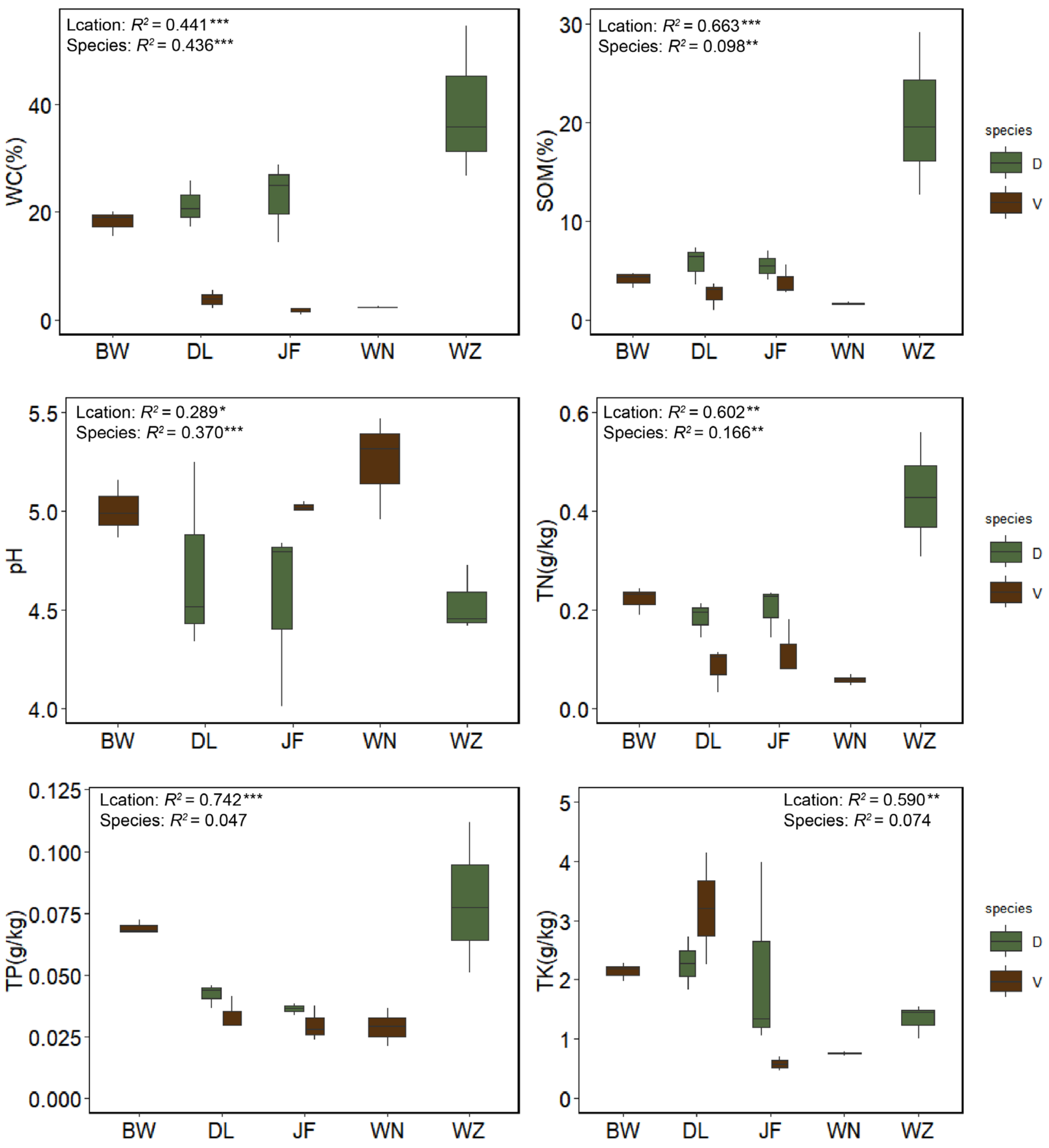

Figure S4 Environmental variables in soil physicochemical properties of *D. pectinatum* and *V. mangachapoi* among different sampling sites. The significant differences between species and sites were detected by adonis. SOM: organic matter, TN: Total nitrogen, TP: Total phosphorus, TK: Total potassium, WC: Water content. *, $P < 0.05$; **, $P < 0.01$, ***, $P < 0.001$.

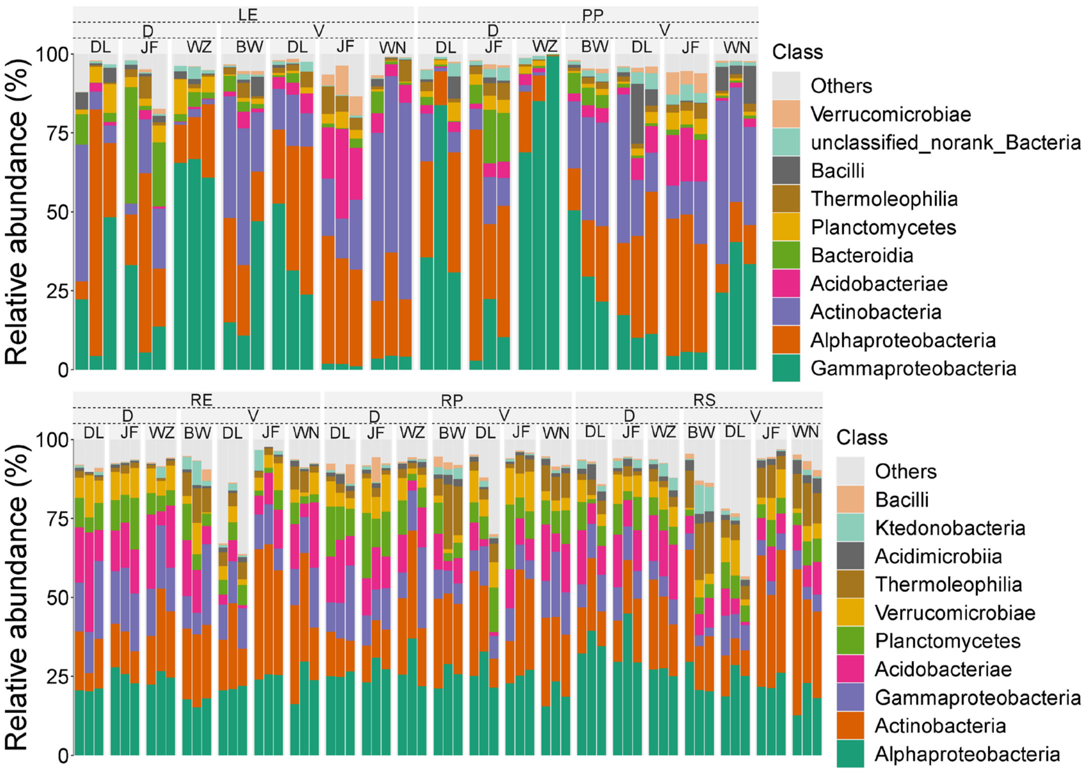

Figure S5 Bacterial community composition at class level of *D. pectinatum* and *V. mangachapoi*.

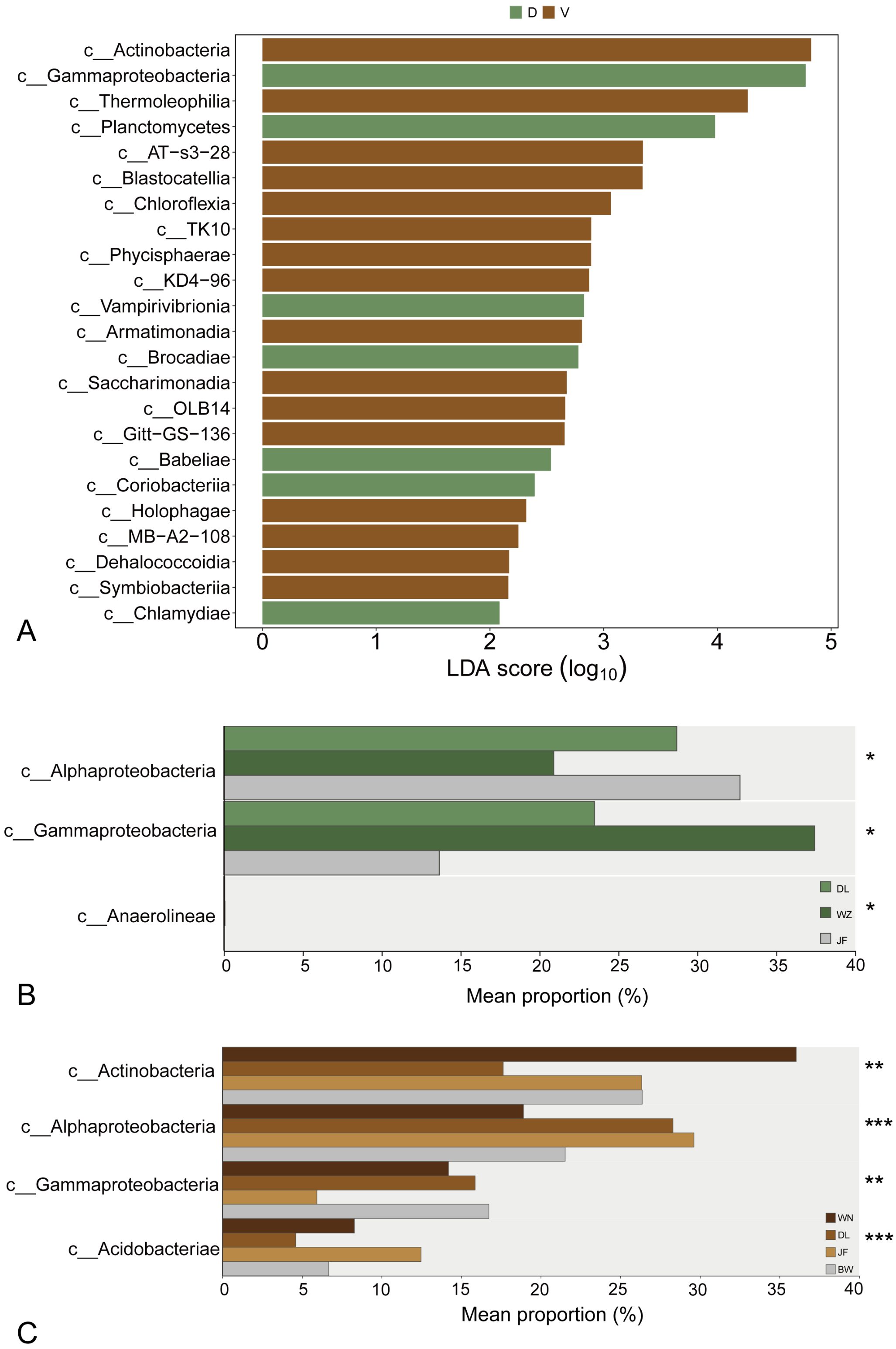

Figure S6 The biomarker taxa of bacterial communities in *D. pectinatum* and *V. mangachapoi*. A: LEfSe identifying the biomarker taxa associated with *D. pectinatum* and *V. mangachapoi* (class level). B and C: Differences in bacterial composition in different geographical locations by One-Way ANOVA in *D. pectinatum* and *V. mangachapoi*, respectively. *, $P < 0.05$; **, $P < 0.01$, ***, $P < 0.001$.

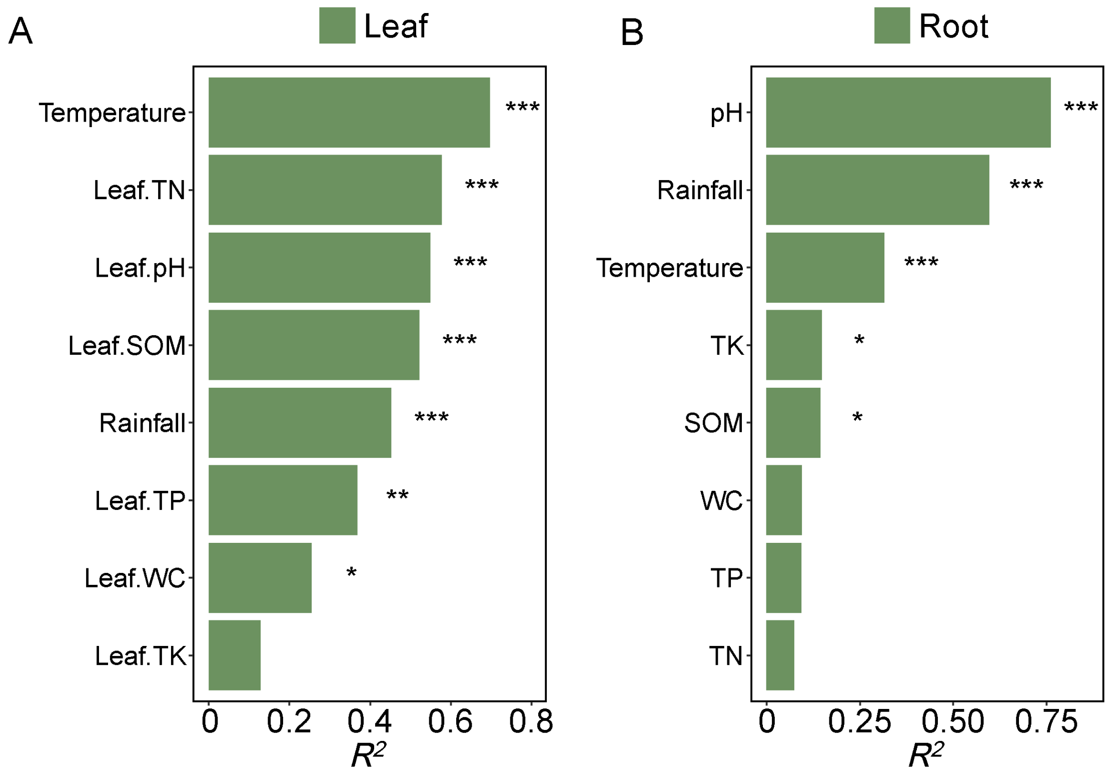

Figure S7 Redundancy analysis ordination of the leaf and soil samples, based on the bacterial community compositions (OTU level). *, $P < 0.05$; **, $P < 0.01$, ***, $P < 0.001$. (A) Leaf sample. (B) Root sample.

Table S1 Permuted multivariate analysis of variance (PERMANOVA) tables for differences in bacterial community compositions (OTU level).

Factors	F	R^2	P
**All**			
Location	2.418	0.044	0.001
Compartment	17.938	0.324	0.001
Species	3.064	0.014	0.004
**LE**			
Location	2.655	0.343	0.001
Species	4.127	0.133	0.001
**PP**			
Location	5.103	0.471	0.001
Species	6.402	0.148	0.001
**RE**			
Location	1.885	0.281	0.005
Species	3.598	0.134	0.001
**RP**			
Location	1.725	0.287	0.004
Species	2.200	0.092	0.016
**RS**			
Location	1.854	0.267	0.013
Species	4.441	0.160	0.001

Table S2 Analysis of variance model results on the effects of geographical locations, plant compartments and plant identity on α-diversity (OTU richness) of bacteria in *D. pectinatum* and *V. mangachapoi*.

**Dataset**	**Variables**	**Pseudo F**	**p-value**
ALL	Compartment	103.430	<0.001
	Species	0.366	0.547
	Location	4.047	<0.01
Phyllosphere	Species	0.075	>0.05
Leaf.endosphere	Species	0.291	>0.05
Root.endosphere	Species	8.588	**<0.05**
Rhizoplane	Species	0.381	>0.05
Rhizosphere	Species	2.328	>0.05
Phyllosphere	Location	1.586	>0.05
Leaf.endosphere	Location	7.296	**<0.01**
Root.endosphere	Location	5.158	**<0.01**
Rhizoplane	Location	0.248	>0.05
Rhizosphere	Location	5.936	**<0.01**
